# Pre-emptive antibiotic therapy to reduce ventilator-associated pneumonia: “thinking outside the box”

**DOI:** 10.1186/s13054-016-1472-5

**Published:** 2016-09-29

**Authors:** Donald E. Craven, Jana Hudcova, Yuxiu Lei, Kathleen A. Craven, Ahsan Waqas

**Affiliations:** 1 Center for Infectious Diseases & Prevention, Lahey Hospital and Medical Center, 31 Mall Rd, Burlington, MA 01805 USA; 2 Surgical Critical Care, Lahey Hospital and Medical Center, Burlington, MA USA; 3 Pulmonary and Critical Care Medicine, Lahey Hospital and Medical Center, Burlington, MA USA; 4 Tufts University School of Medicine, Boston, MA USA; 5 New England Independent Review Board for Human Research, Wellesley, MA USA; 6 Brookdale University Medical Center, Brooklyn, NY USA

**Keywords:** Ventilator-associated respiratory infection (VARI), Ventilator-associated tracheobronchitis (VAT), Ventilator-associated pneumonia (VAP), Bacterial pathogen virulence, Antibiotic sensitivity, Use of early appropriate antibiotic therapy, VAP prevention, Patient outcomes, Morbidity, Mortality and healthcare costs

## Abstract

Mechanically ventilated, intubated patients are at increased risk for tracheal colonization with bacterial pathogens that may progress to heavy bacterial colonization, ventilator-associated tracheobronchitis (VAT), and/or ventilator-associated pneumonia (VAP). Previous studies report that 10 to 30 % of patients with VAT progress to VAP, resulting in increased morbidity and significant acute and chronic healthcare costs. Several natural history studies, randomized, controlled trials, and a meta-analysis have reported antibiotic treatment for VAT can reduce VAP, ventilator days, length of intensive care unit (ICU) stay, and patient morbidity and mortality. We discuss early diagnostic criteria, etiologic agents, and benefits of initiating, early, appropriate intravenous or aerosolized antibiotic(s) to treat VAT and reduce VAP, to improve patient outcomes by reducing lung damage, length of ICU stay, and healthcare costs.

## Background

Ventilator-associated respiratory infections (VARI) often begin with bacterial colonization that may progress to include ventilator-associated tracheobronchitis (VAT) and ventilator-associated pneumonia (VAP) diagnosed after 48 h of intubation [1–10]. VAT and VAP have similar clinical signs of fever, leukocytosis, purulent secretions, and positive bacterial cultures, except VAP requires a new infiltrate on chest radiograph. Rates of progression from VAT to VAP range between 7 and 30 % [3–7]. VAP mortality rates are 20 to 50 % with healthcare costs of $20,000 to $40,000 per patient. Inappropriate antibiotic therapy for VAP increases patient mortality (*p* = 0.001), hospital mortality (*p* < 0.0001), ventilator days (16 versus 7, *p* < 0.0005), intensive care unit (ICU) days (14 versus 8, *p* = 0.02), and hospital days (42 versus 28, *p* = 0.04) [4].

VAT is an unappreciated but important early clinical condition in ventilated patients which has been linked to VAP, resulting in significant patient morbidity and mortality [3–7]. The incidence of VAT ranges from 2.7 to 11.5 % in randomized trials and 7 to 30 % of VAT patients will progress to VAP. Gram-negative pathogens are responsible for >75 % of the episodes and virulence is greater for *Pseudomonas aeruginosa* isolates with toxins or other multi-drug resistant (MDR) pathogens that require early, effective, intravenous or aerosolized antibiotic therapy, as well as re-evaluation of non-responders to antibiotic therapy.

Several studies have compared outcomes of ventilated patients, focusing on increased ventilator days and length of ICU stay for VAT and VAP patients. In addition, numerous studies have demonstrated improved patient outcomes in VAT patients treated with appropriate antibiotics [3–9]. Nseir et al. [6] studied 58 VAT patients randomly assigned to intravenous antibiotic therapy (*n* = 22) versus no therapy (*n* = 36). The antibiotic-treated group had more mechanical ventilation-free days (12 versus 2, *p* < 0.001), reduced VAP (13 versus 47 %, *p* = 0.01), and significantly lower ICU mortality (18 versus 47 %, *p* = 0.047).

Aerosolized antibiotic therapy for VAT and VAP has attracted increased interest. It is administered using an in-line nebulizer and has the advantage over intravenous therapy of being able to deliver a higher concentration of antibiotics into all parts of the lung, which can decrease bacterial lung burden, pulmonary damage and associated complications, as well as *Clostridium difficile* colitis resulting from intravenous antibiotic therapy. Of note, Palmer and Smaldone [9] reported decreased respiratory infections in VAT patients treated with aerosolized antibiotics; 26 of 27 organisms were eradicated compared with placebo (*p* < 0.0001) and 14 out of 16 patients were cured (*p* < 0.001).

Serial endotracheal aspirate (ETA) surveillance samples can help clinicians identify numbers of pathogens and the antibiotic sensitivity profile needed to initiate earlier appropriate antibiotic therapy to decrease colonization, VAT, and VAP [3–8]. In “thinking outside the box”, serial ETA “surveillance cultures” can be a valuable clinical tool to monitor the levels of airway colonization with bacterial pathogens and facilitate use of pre-emptive, targeted antibiotic therapy for VAT to reduce VAP, ventilator days, lung bacterial burden, and risk of post-traumatic stress disorder or delirium [4–13].

Depuydt and coworkers [8] demonstrated the benefit of serial ETA surveillance cultures for identifying MDR pathogens and earlier appropriate antibiotic therapy for VAP rather than empiric broad spectrum antibiotics with de-escalation. However, the increased availability of rapid microbiologic diagnostic methods for identifying and treating specific pathogens and the use of antibiotic sensitivity profiles of bacteria in ETA samples—using “matrix-assisted laser desorption ionization mass spectrometry (MALDI-TOF), which can facilitate early, appropriate pre-emptive antibiotic therapy for VAT and/or VAP—will improve patient outcomes and reduce healthcare costs.

Ventilated ICU patients are invariably complicated, often elderly, critically ill patients with underlying acute and or chronic diseases or post-surgery and who are at increased risk for VARI, an “infection vortex” with complications such as lung bacterial burden, damage that increases host debility, and delirium or post traumatic stress disorder, which may linger for months or years, resulting in hospital readmissions and enormous healthcare costs [12–14]. For example, Unroe et al. [14] studied 99 ventilated patient survivors from five ICUs at Duke who were carefully followed for one year after discharge. These patients underwent 150 hospital readmissions and multiple transitions of care between acute, chronic, and long-term care or rehabilitation. Of note is that one year following hospital discharge, only 9 % of the 99 study patients were living independently with estimated costs of $3.5 million per survivor. All of these poor outcomes underscore the importance of early and effective antibiotic therapy to reduce lung bacterial burden and to make an effort to improve patient outcomes in ventilated patients.

## Conclusions

We recommend use of VAP prevention strategies with a focus on earlier diagnosis and use of pre-emptive, appropriate antibiotic therapy based on clinical signs and microbiologic evidence of heavy endotracheal colonization, VAT, or VAP. This can be done by using serial lung surveillance cultures to identify bacterial pathogens and antibiotic sensitivity profiles, assessing levels of bacterial colonization for the presence of more virulent strains, such as *Staphylococcus aureus* or *P. aeruginosa*, which produce toxins, or MDR Gram-negative bacterial pathogens. Ventilated patients should undergo careful monitoring to assess the need for early antibiotic therapy and so the endotracheal tube is removed as soon as possible to reduce leakage around the endotracheal tube cuff or biofilm emboli from the endotracheal tube lumen. Careful monitoring is also needed to assess the impact of early antibiotic therapy, the use of sedation vacations, and “walk to wean” programs for better patient outcomes and reduced hospital stay and associated healthcare costs. In 1895, Sir William Osler MD, an expert on pneumonia, said “Remember how much you don’t know”. Today, he might add “Remember how much your care team know and can apply.”

## Abbreviations

ETA: Endotracheal aspirate
ICU: Intensive care unit
MDR: Multi-drug resistant
VARI: Ventilator-associated respiratory infections
VAP: Ventilator-associated pneumonia
VAT: Ventilator-associated tracheobronchitis
